# Deciphering tumor immune microenvironment differences between high-grade serous and endometrioid ovarian cancer to investigate their potential in indicating immunotherapy response

**DOI:** 10.1186/s13048-023-01284-1

**Published:** 2023-11-22

**Authors:** Hua Yang, Xiangyu Gu, Rong Fan, Qun Zhu, Sen Zhong, Xirun Wan, Qian Chen, Lan Zhu, Fengzhi Feng

**Affiliations:** 1 Department of Obstetrics and Gynecology, Peking Union Medical College Hospital, National Clinical Research Center for Obstetric & Gynecologic Diseases, Chinese Academy of Medical Sciences & Peking Union Medical College, No.1 Shuai Fu Yuan, Wang Fu Jing Street, Beijing, China; 2 4+4 Medical Doctor Program, Chinese Academy of Medical Sciences & Peking Union Medical College, Beijing, China; 3 Department of Obstetrics and Gynecology, Beijing Puren Hospital, Beijing, China; 4 Thorgene Co., Ltd, Beijing, China

**Keywords:** Ovarian epithelial carcinoma, Immunotherapy, Sequence analysis, DNA mutational analysis, Immune microenvironment

## Abstract

**Background:**

Ovarian cancer is a significant public health concern with a poor prognosis for epithelial ovarian cancer. To explore the potential of immunotherapy in treating epithelial ovarian cancer, we investigated the immune microenvironments of 52 patients with epithelial ovarian cancer, including 43 with high-grade serous ovarian cancer and 9 with endometrioid ovarian cancer.

**Results:**

Fresh tumor tissue was analyzed for genetic mutations and various parameters related to immune evasion and infiltration. The mean stromal score (stromal cell infiltration) in high-grade serous ovarian cancer was higher than in endometrioid ovarian cancer. The infiltration of CD8 T cells and exhausted CD8 T cells were found to be more extensive in high-grade serous ovarian cancer. Tumor Immune Dysfunction and Exclusion scores, T cell exclusion scores, and cancer-associated fibroblasts (CAF) scores were also higher in the high-grade serous ovarian cancer group, suggesting that the number of cytotoxic lymphocytes in the tumor microenvironment of high-grade serous ovarian cancer is likely lower compared to endometrioid ovarian cancer.

**Conclusions:**

The high mean stromal score and more extensive infiltration and exhaustion of CD8 T cells in high-grade serous ovarian cancer indicate that high-grade serous ovarian cancer exhibits a higher level of cytotoxic T cell infiltration, yet these T cells tend to be in a dysfunctional state. Higher Tumor Immune Dysfunction and Exclusion scores, T cell exclusion scores, and CAF scores in high-grade serous ovarian cancers suggest that immune escape is more likely to occur in high-grade serous ovarian cancer, thus endometrioid ovarian cancer may be more conducive to immunotherapy. Therefore, it is crucial to design immunotherapy clinical trials for ovarian cancer to distinguish between high-grade serous and endometrioid ovarian cancer from the outset. This distinction will help optimize treatment strategies and improve outcomes for patients with different subtypes.

## Background

Ovarian cancer (OC) is a major type of malignant tumors affecting the female reproductive system, with epithelial ovarian cancer (EOC) being the most common subtype, accounting for over 95% of ovarian malignancies [1, 2]. The standard treatment for EOC involves cytoreductive surgery and platinum/taxane chemotherapy. In recent years, the emergence of PARP inhibitors has significantly improved progression-free survival (PFS) for patients with EOC [3, 4]. However, the clinical benefit of available treatments remains limited, with 5-year survival rates for ovarian cancer remaining below 50% after diagnosis [5]. Hence there is a continued need for new therapies, identification of patients who would benefit most from these therapies, and the development of optimal therapeutic strategies [6, 7].

The field of cancer immunotherapy has experienced remarkable advancements in recent years, particularly with the success of immune checkpoint inhibitors in treating several types of malignancies such as melanoma, renal cell carcinoma, bladder cancer, non-small cell lung carcinoma, and Hodgkin’s disease [8–10]. However, the clinical use of checkpoint inhibitors in ovarian cancer has shown limited success, with the single-agent objective response rates in clinical trials ranging around 10–15%. These suboptimal response rates may be attributed to the unique pathological characteristics of ovarian cancer, genetic mutations, and the distinct features of the immune microenvironment [11]. EOC is classified into five major subtypes, including high-grade serous (HGSOC), low-grade serous, clear cell, endometrioid (EEOC), and mucinous ovarian cancer [12]. Sensitivity to platinum-based chemotherapy varies among these subtypes. Clinical trials often group HGSOCs and EEOCs together due to their clinical evidence of high sensitivity to platinum-based chemotherapy. However, it remains unclear whether there are differences in the response to targeted therapy and immunotherapy among histological subtypes, particularly between HGSOCs and EEOCs. The lack of successful immunotherapy strategies has prompted this study to comprehensively analyze whole exome DNA sequence information and transcriptome sequencing data from histologically confirmed HGSOCs and EEOCs samples in order to identify cases that may be potentially suitable for immunotherapy.

## Methods

### Study design and patient population

In this study, fresh tissue and peripheral blood were prospectively collected from 57 patients with ovarian cancer between October 2018 and July 2020, including 43 patients with high-grade serous ovarian cancer (HGSOC), 9 with endometrioid ovarian cancer (EEOC), 2 with clear cell carcinoma, 2 with mucous cystadenocarcinoma, and 1 with low-grade serous cystadenocarcinoma. All patients underwent standard cytoreductive surgery followed by adjuvant chemotherapy with carboplatin (area under the curve [AUC] = 5)/paclitaxel (175 mg/m^2^), as per the treatment protocol of the experienced oncologist team. The follow-up period extended until December 2022 to make sure a minimum of 1-year follow-up for all patients. This study was approved by the Institutional Review Board of Peking Union Medical College Hospital (JS-1936).

The inclusion criteria for patients were as follows: [1] histologically confirmed HGSOC or EEOC, [2] underwent cytoreductive surgery, [3] received first-line adjuvant chemotherapy with at least 6 cycles of carboplatin/paclitaxel, [4] progression-free survival (PFS). PFS was defined as the duration from the end of the last chemotherapy treatment to the date of first disease recurrence. Cytoreductive surgery outcomes were divided into R0 (No visual residual lesions), R1 (≤ 1 cm residual disease), and R2 (> 1 cm residual disease). The patient’s response to chemotherapy and disease recurrence were assessed based on RECIST criteria or CA125 progression criteria as defined by the Gynecological Cancer InterGroup [13].

### Sample collection and storage

Tumor tissues obtained during surgery were collected with tubes containing nucleic acid protection solution, and paired peripheral blood samples were collected using EDTA tubes. If the experiment can’t be performed immediately, all the samples were snap-frozen in liquid nitrogen within 30 min after resection.Genomic DNA was extracted from all included samples. The matched peripheral blood leukocytes were used as the source for germline DNA control.

### Whole-exome sequencing (WES) and somatic mutation calling

Tumor and matched normal DNA were extracted using the TIANamp Genomic DNA Kit (DP304, TIANGEN, Beijing, China) from fresh tumor tissue and paired blood sample according to the manufacturer’s recommendations. Libraries were constructed by the Agilent SureSelectXT Human All Exon V6 Kit (5190–8864, Agilent Technologies, Santa Clara, USA) and sequenced with next-generation sequencing. Genomic DNA was fragmented, end-repaired, adenylated at the 3’ ends, end-connected, amplified, purified, and size-selected in the process of library construction, then was sequenced on the Illumina X10 platform (Illumina Inc., San Diego, CA, USA).

The whole-exome sequencing (WES) depth of tumor samples is > 200× and the WES depth of normal samples is > 100×. After removing sequencing reads containing adaptor sequences and low-quality reads, which have too many Ns (> 5%) and low-quality bases (> 15% bases with quality ≤ 19), high-quality paired-end reads were mapped to the reference genome (human genome build, hg19) by Burrows-Wheeler Aligner (BWA version 0.7.15, BWA-MEM algorithm). Then somatic SNVs and InDels were analyzed via GATK MuTect2 (version 4.1).

### RNA-seq and gene expression

Tumor RNA was extracted using the TRIzol® Reagent (15596018, Invitrogen, Burlington, USA) from fresh tissue. Libraries were constructed using a NEBNext® UltraTM II RNA Library Prep Kit (#E7775, NEB, MA, USA) according to the manufacturer’s recommendations, and sequenced with NGS. Total RNA was fragmented, reverse transcribed into complementary DNA, base ‘A’ added in the 3’ ends, adapter connected, amplified and purified, and then sequenced on Illumina X10 platform (Illumina Inc., San Diego, CA, USA).

Raw sequence reads were quality controlled using filter pipeline with multiple filtering steps as follows: [1] removing reads with adapters; [2] removing reads in which unknown bases were more than 5%; [3] removing reads in which more than 15% of bases had low quality (sequencing quality no more than 19). After filtering, the remaining high-quality clean reads were retained for downstream bioinformatic analysis. High-quality paired-end reads were mapped to hg19 using Bowtie2 (version 2.2.4) software from Tophat2 (version 2.0.10) with default parameters. The program Cufflinks (version 2.2.1) was used to calculate the expression levels of genes in terms of reads per kilobases per million reads (FPKM).

### Assessment of immune infiltration and immune checkpoints

Based on the gene expression (FPKM), the ESTIMATE algorithm [14] was used to evaluate the fraction of stromal and immune cells in tumor samples. ESTIMATE outputs ‘Stromal score’ and ‘Immune score’ based on the gene signatures related to stromal cell and immune cell infiltration. Besides, unsupervised clustering of immune signatures by Danaher [15] was performed to identify the immune infiltration of different cell subtypes.

To analyze pathway-level enrichment rather than individual genes, we applied Single-Sample Gene Set Enrichment Analysis (ssGSEA) [16], which calculates enrichment scores for sample-gene set pair and allows clustering based on pathways. Using ssGSEA, we measured the T cell infiltration score (TIS) and overall immune infiltration score (IIS) based on individual cell population metrics [17]. TIS was an aggregate score based on the mean of the standardized values of nine T-cell subsets: CD8 T, T helper, T, T central and effector memory, Th1, Th2, Th17, and Treg cells. IIS was defined as the mean of the standardized values for macrophages, DC subsets (total, plasmacytoid, immature, activated), B cells, cytotoxic cells, eosinophils, mast cells, neutrophils, NK cell subsets (total, CD56bright, CD56dim), and all T-cell subsets used in the computation of TIS. CD8 + T cell exhaustion score [18], APM score [17], angiogenesis score [17, 19], and TGFβ score [20] were also calculated with the ssGSEA algorithm.

Furthermore, normalized gene expression data was used for the evaluation of tumor immune dysfunction and exclusion (TIDE) [21].

### Estimation of tumor mutational burden (TMB) and intratumoral heterogeneity

TMB value was calculated based on the total number of somatic mutations per the whole exon length (mutations/Mb). Based on the MATH algorithm, the intratumor heterogeneity of each patient was evaluated [22]. Decomposition of mutational signatures was performed using the R package ‘deconstructSigs’ [23], based on the set of 30 mutational signatures (‘COSMIC-signature.v2’, https://cancer.sanger.ac.uk/cosmic/signatures_v2) for all these samples, which were analyzed on the basis of the SNVs and their sequence context, considering the immediately flanking 5’and 3’nucleotides. Mutational signatures with ≥ 0.08 weight in each case were considered to have a substantial contribution to the mutational landscape of a sample.

### Statistical analysis

Continuous variables were compared with unpaired t test or Mann-Whitney U test. Categorical variables were compared with Fisher’s exact test. The Log-rank test was used to generate P values in survival analysis. All correlation analyses were performed using the Spearman correlation analysis. In all analyses, a two-sided or two-tailed P value < 0.0500 was considered statistically significant. Effect size was represented by the absolute value of Cohen’s d (unpaired t test), Cohen’s w (Fisher’s exact test) and Rank-Biserial correlation coefficient r (Mann-Whitney U test). Gene mutation landscape was plotted with the R package ‘ComplexHeatmap’ (version 2.12.1). The histogram of mutational signatures was plotted with the R package ‘ggplot2’ (version 3.4.1). Effect size was estimated with the R package ‘effectsize’ (version 0.8.5). All the scatter diagrams between two groups were drawn using GraphPad Prism 8.0 software (San Diego, USA).

## Results

### Patients’ clinical characteristics and gene mutation profiles

A total of 52 patients who met the study’s inclusion/exclusion criteria were included in the data analysis. Among these patients, 43 were diagnosed with HGSOC and 9 were diagnosed with EEOC. The clinical staging of the cases ranged from stage I to IV, including 2 cases in stage I, 5 cases in stage II, 38 cases in stage III, and 7 cases in stage IV. The age of the 52 patients ranged from 34 to 80 years, with a median age of 56 years. Preoperative CA125 values of 52 patients varied from 20.6 to 6611.0 IU/ml, with a median value of 715.5 IU/ml (Table 1).

**Table 1:** Clinical characteristics of patients with either of two pathological subtypes

Characteristic	All cases (n = 52)	HGSOC (n = 43)	EEOC (n = 9)	P-value	Effect size
Age, median (range), years	56 (34–80)	54 (34–80)	62 (42–76)	0.3593	0.34
Stage, %					
I-II	7	4 (9.30%)	3 (33.33%)	0.0900	1.00
III-IV	45	39 (90.70%)	6 (66.67%)		
Pre_CA125, median (range), IU/ml	715.5 (20.6–6611.0)	748.0 (20.6–6611.0)	430.9 (220.7–1902.0)	0.4173	0.18
PFS, median (range), months		20 (2–44)	undefined (3–43)	0.5230	
Recurrence, %					
Recurrence	24	20 (46.51%)	4 (44.44%)	> 0.9999	1.00
No recurrence	28	23 (53.49%)	5 (55.56%)		
Treated with PARP inhibitor, %					
Yes	21	20 (46.51%)	1 (11.11%)	0.0670	1.00
No	31	23 (53.49%)	8 (88.89%)		
Surgical excision, %					
CRS (R0)	28	21 (48.84%)	7 (77.78%)	0.3366	1.00
CRS (R1)	22	20 (46.51%)	2 (22.22%)		
CRS (R2)	2	2 (4.65%)	0 (0.00%)		

Footnote: HGSOC, high-grade serous ovarian cancer. EEOC, endometrioid epithelial ovarian cancer. Pre_CA125, preoperative serum carbohydrate antigen 125 values. PFS, progression-free survival. PARP, poly ADP-ribose polymerase. CRS, cytoreductive surgery. R0, no macroscopic residual disease. R1, 1–10 mm residuals. R2, > 10 mm residuals. Statistics in Age: unpaired t test; Statistics in Pre_CA125: Mann–Whitney U test; Statistics in Stage, Recurrence, Treated with PARP inhibitor and Surgical excision: Fisher’s exact test; Statistics in PFS: Log-rank test

**Fig. 1:**
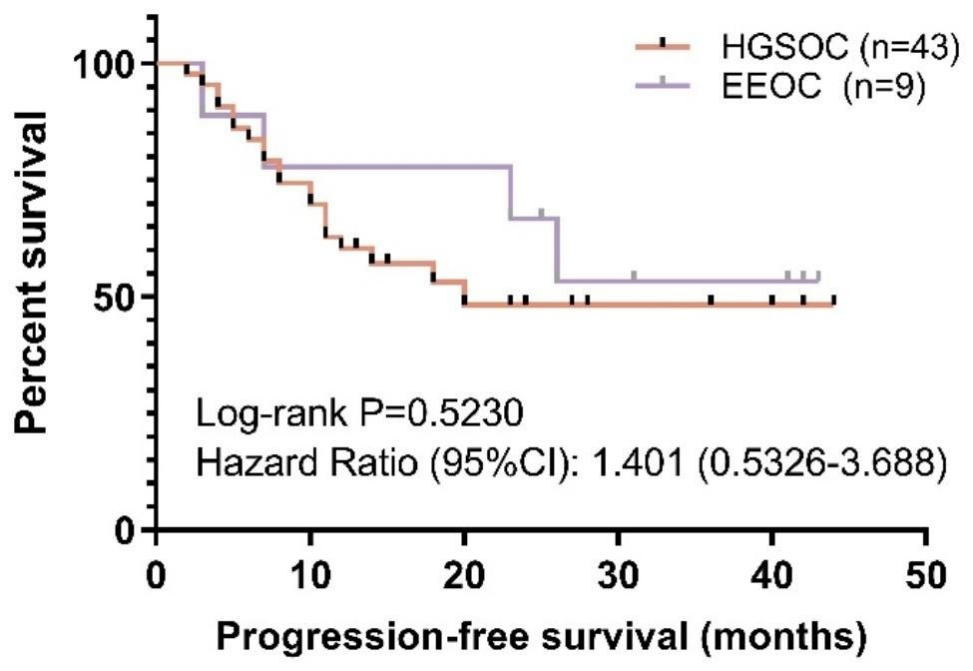
Comparison of Progression-Free Survival between the two subtypes. HGSOC, high-grade serous ovarian cancer. EEOC, endometrioid epithelial ovarian cancer. Statistics: Log-rank test

Additionally, a survival analysis was performed to compare PFS between the two subtypes. The median PFS for HGSOC was 20 months, while it was undefined for EEOC. The results revealed that there is no statistically significant difference in PFS between the two histotypes (Log-rank P = 0.5230; Hazard Ratio = 1.401) (Fig. 1).

**Fig. 2:**
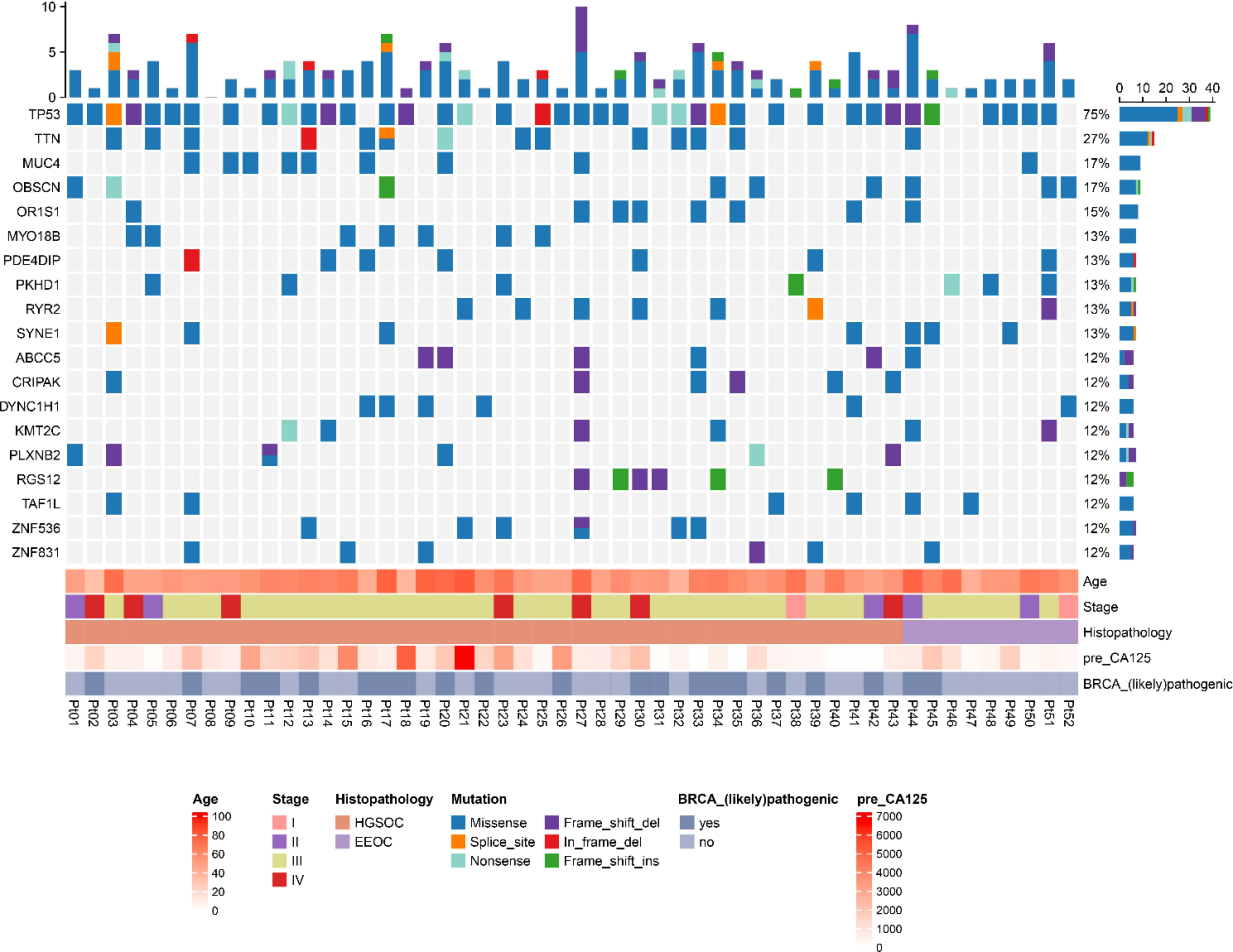
Gene mutation spectrum and clinical characteristics of 52 ovarian cancer patients. pre-CA125, Preoperative serum carbohydrate antigen 125. pre_CA125, preoperative serum carbohydrate antigen 125. HGSOC, high-grade serous ovarian cancer. EEOC, endometrioid epithelial ovarian cancer

A total of 6328 mutation sites were identified in the tumor tissues, with a median of 101 mutations (range: 11–582 mutations). The most frequently mutated gene was TP53, with a mutation frequency of 75.47% (39/52). The overall tumor mutation burden (TMB) was moderate, with a median value of 1.5538 (ranging from 0.1692 to 8.9538). Among the 52 patients, 21(40%) patients were detected with deleterious BRCA mutations (Fig. 2).

### Differences in gene mutation characteristics between HGSOC and EEOC

Among the 52 patients, 19 genes were found to have a mutation frequency greater than 10% (mutations in at least 6 samples) (Table 2). For these 19 genes, the mutation frequency in 43 HGSOC patients ranged from 9.30 to 76.74%, while from 0.00 to 66.67% in 9 EEOC patients. TP53 was the most frequently mutated gene in both HGSOC (76.74%) and EEOC patients (66.67%). However, there was no significant difference in the number of mutated genes between the two subtypes (Table 2).

**Table 2:** Differences in the number of samples with specific mutations between the two pathological subtypes

Gene	HGSOC (n = 43)	EEOC (n = 9)	P-value	Effect size
TP53	33 (76.74%)	6 (66.67%)	0.6739	1.00
TTN	13 (30.23%)	1 (11.11%)	0.4147	1.00
MUC4	8 (18.60%)	1 (11.11%)	> 0.9999	1.00
OBSCN	6 (13.95%)	3 (33.33%)	0.1767	1.00
OR1S1	7 (16.28%)	1 (11.11%)	> 0.9999	1.00
RYR2	6 (13.95%)	1 (11.11%)	> 0.9999	1.00
ABCC5	5 (11.63%)	1 (11.11%)	> 0.9999	1.00
MYO18B	7 (16.28%)	0 (0.00%)	0.3309	1.00
PDE4DIP	6 (13.95%)	1 (11.11%)	> 0.9999	1.00
PKHD1	4 (9.30%)	3 (33.33%)	0.0900	1.00
SYNE1	4 (9.30%)	3 (33.33%)	0.0900	1.00
ZNF536	6 (13.95%)	0 (0.00%)	0.5745	1.00
ZNF831	5 (11.63%)	1 (11.11%)	> 0.9999	1.00
CRIPAK	6 (13.95%)	0 (0.00%)	0.5745	1.00
DYNC1H1	5 (11.63%)	1 (11.11%)	> 0.9999	1.00
KMT2C	4 (9.30%)	2 (22.22%)	0.2750	1.00
PLXNB2	6 (13.95%)	0 (0.00%)	0.5745	1.00
RGS12	6 (13.95%)	0 (0.00%)	0.5745	1.00
TAF1L	4 (9.30%)	2 (22.22%)	0.2750	1.00

Footnote: HGSOC, high-grade serous ovarian cancer. EEOC, endometrioid epithelial ovarian cancer. Statistics: Fisher’s exact test

**Fig. 3:**
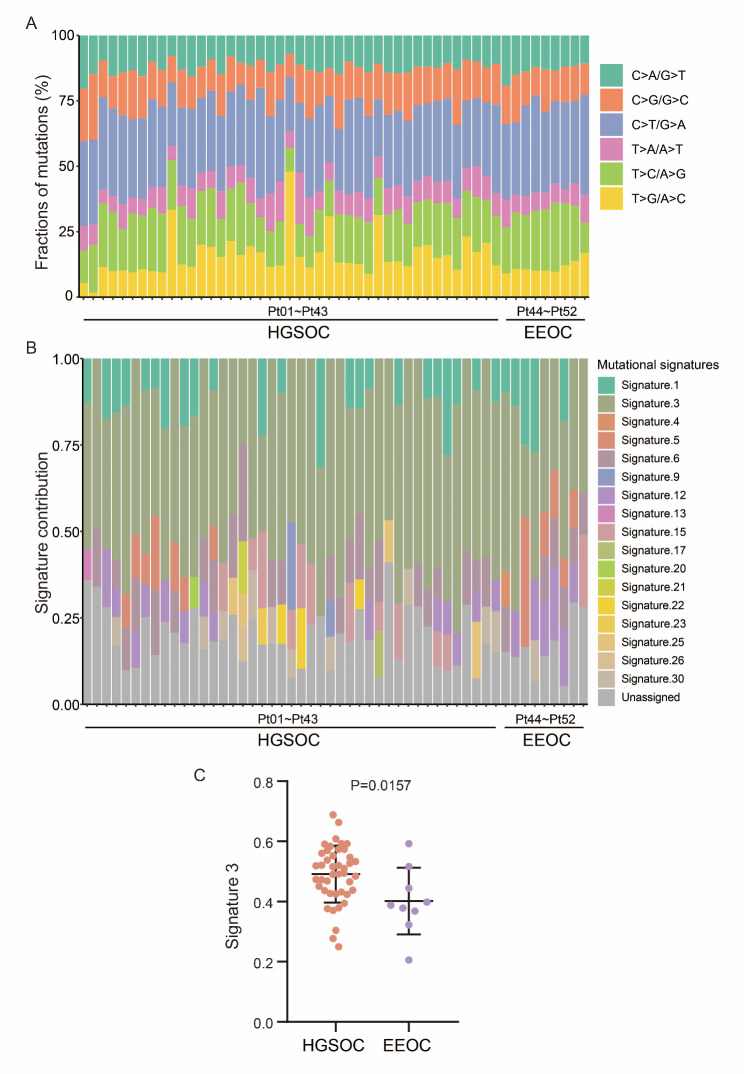
Mutational signatures of HGSOC and EEOC patients. **A,** six different single nucleotide substitutions were detected in HGSOC and EEOC. **B,** proportions of somatic mutations in 17 mutation signatures for each patient. **C,** the contribution of signature 3 in HGSOC patients and EEOC patients. HGSOC, high-grade serous ovarian cancer. EEOC, endometrioid epithelial ovarian cancer. Data in C was shown as ‘Mean with SD’. Statistics in C: unpaired t test

Additionally, single-base mutation analysis revealed that C > T/G > A mutations were the dominant form of single-base mutations in the 52 patients with EOC (Fig. 3A). A total of 17 mutation signatures were detected, with signature 3 having the highest weight proportion (Fig. 3B). Statistically, the weight of signature 3 in HGSOC patients was significantly higher than in EEOC patients (average, 0.4908 vs. 0.4016; P = 0.0157; Cohen’s d = 0.92; Fig. 3C). Signature 3 is associated with the failure to repair DNA double-strand breaks by homologous recombination.

Furthermore, the tumor mutation burden (TMB) was compared between the two subtypes. The results showed that the mean TMB for HGSOC was 1.8655 (range, 0.1692–8.9538), while the mean TMB for EEOC was 1.9043 (range, 0.4462–5.1077). However, this difference in TMB between the two subtypes was not statistically significant (P = 0.6214, Rank-Biserial correlation r = 0.11, Mann-Whitney U test). Additionally, tumor heterogeneity was evaluated in both subtypes. The mean tumor heterogeneity for HGSOC was 50.5965 (range, 0.0000-89.1367), and for EEOC was 42.7625 (range, 19.0999-78.9000). Nevertheless, there is no difference in tumor heterogeneity between the two subtypes (P = 0.1549, Rank-Biserial correlation r = 0.31, Mann-Whitney U test), which might be attributed to the relatively small sample size of EEOC in our study.

### Differences of immune infiltration in tumor tissues of HGSOC and EEOC

**Fig. 4:**
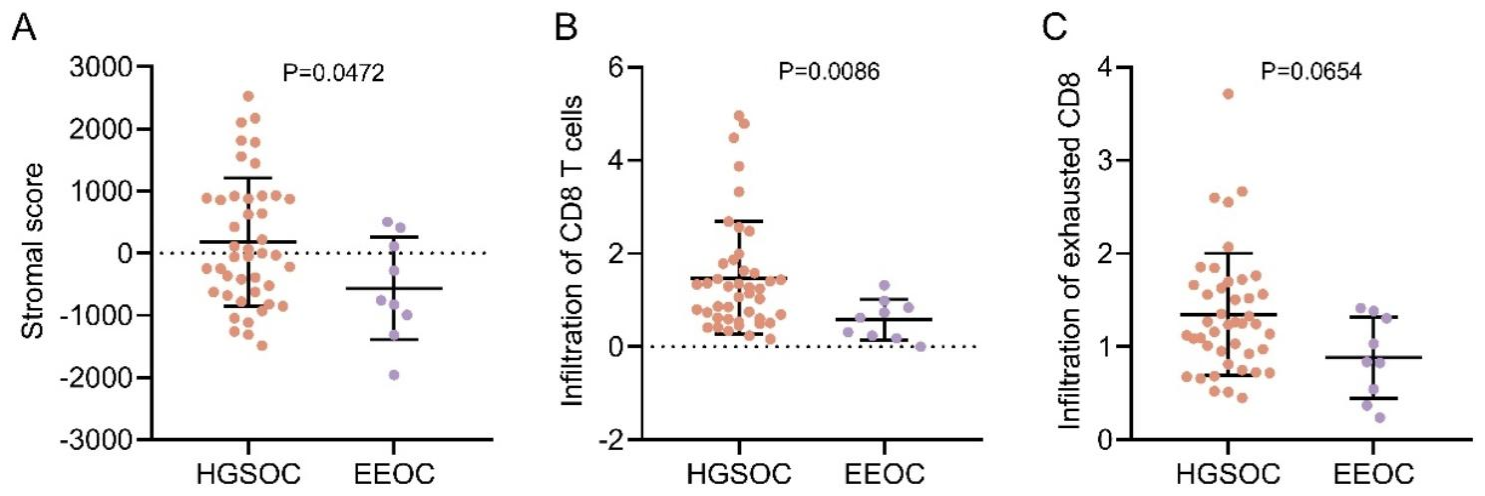
Comparison of immune infiltration between the two subtypes. **A,** Stromal score ([5]). **B-C**, CD8 T cell and exhausted CD8 T cell infilteration (Danaher[7]). HGSOC, high-grade serous ovarian cancer. EEOC, endometrioid epithelial ovarian cancer. Data was shown as ‘Mean with SD’. Statistics in **A:** unpaired t test; Statistics in **B-C:** Mann–Whitney U test

The immune infiltration in tumor tissues was evaluated using the ESTIMATE algorithm. The results revealed that the mean stromal score for HGSOC ranged between − 1485.6980 and 2524.6021, with a mean score of 178.6252. The mean stromal score for EEOC ranged between − 1958.7859 and 502.8649, with a mean score of -567.7159. HGSOC had a higher stromal score than EEOC, and the difference between stromal scores was statistically significant (P = 0.0472, Cohen’s d = 0.75) (Fig. 4A). Evaluation of CD8 T cells infiltration using the Danaher algorithm demonstrated that HGSOC exhibited higher levels of CD8 T cell infiltration (ranged from 0.1629 to 4.9603; mean, 1.4733) compared to EEOC (range, 0.0000-1.3144; mean, 0.5790) (P = 0.0086, Rank-Biserial correlation r = 0.55) (Fig. 4B). Furthermore, the level of exhausted CD8 T cell infiltration was also evaluated. The results showed that the mean score for exhausted CD8 T cell infiltration was 1.3441 (range, 0.4505–3.7122) for HGSOC and 0.8819 (range, 0.2375–1.4132) for EEOC. While the mean value in exhausted CD8 T cell infiltration for HGSOC was higher, no statistically significant difference was observed between these two pathological subtypes (P = 0.0654, Rank-Biserial correlation r = 0.40) (Fig. 4C).

**Fig. 5:**
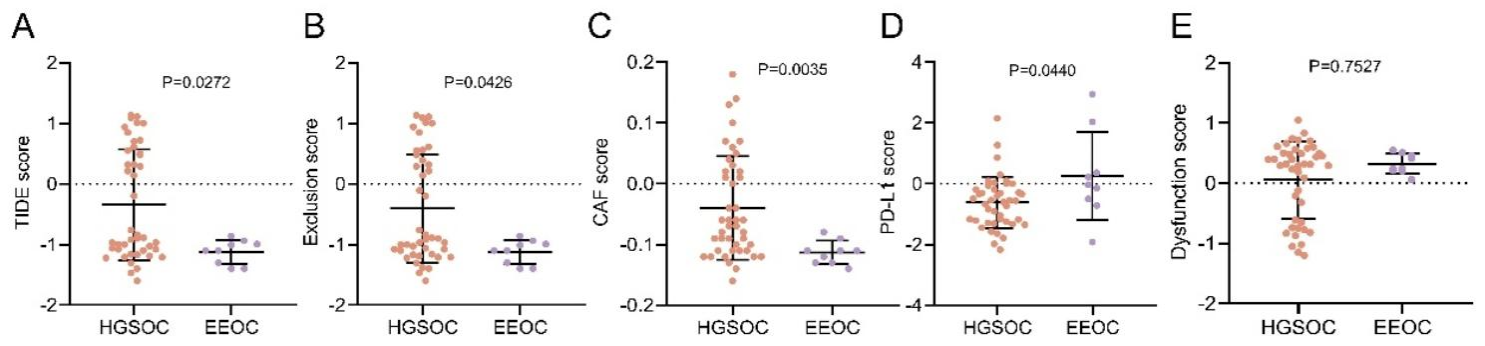
Comparison of immune escape between the two subtypes. **A-D**, TIDE score, exclusion score, cancer-associated fibroblasts (CAF) score and PD-L1 score ([14]). HGSOC, high-grade serous ovarian cancer. EEOC, endometrioid epithelial ovarian cancer. Data was shown as ‘Mean with SD’. Statistics in **A** to **E**: Mann–Whitney U test

To evaluate differences in immune evasion between the two pathological subtypes, transcriptome data were analyzed using the Tumor Immune Dysfunction and Exclusion (TIDE) algorithm. The results demonstrated that the TIDE score ranged from − 1.6000 to 1.1300 (mean: -0.3435) for the HGSOC group and from − 1.4000 to -0.8600 (mean: -1.1222) for EEOC (P = 0.0272, Rank-Biserial correlation r = 0.47, Fig. 5A). The exclusion score for the HGSOC group ranged from − 1.6000 to 1.1300 (mean: -0.4081), while the score for the EEOC group ranged from − 1.4000 to -0.8600 (mean: -1.1222) (P = 0.0426, Rank-Biserial correlation r = 0.43, Fig. 5B). The differences in TIDE and exclusion scores between the two groups were statistically significant. The CAF score (range, HGSOC: -0.1600 to 0.1800, EEOC: -0.1400 to -0.0800; mean, -0.0400 vs. -0.1133, P = 0.0035, Rank-Biserial correlation r = 0.60, Fig. 5C) was significantly higher in the HGSOC group, while the PD-L1 score (range, HGSC: -2.1700 to 2.1500, EEOC: -1.9200 to 2.9400; mean, -0.6156 vs. 0.2478; P = 0.0440; Rank-Biserial correlation r = 0.43; Fig. 5D) was significantly higher in the EEOC group. The differences in dysfunction score (range, HGSOC: -1.2000 to 1.0500, EEOC: 0.0600 to 0.5500; mean, 0.0537 vs. 0.3200; P = 0.7527; Rank-Biserial correlation r = 0.07; Fig. 5E) and TAM M2 (range, HGSOC: -0.0700 to 0.0400, EEOC: -0.0200 to 0.0200; mean, 0.0016 vs. 0.0011; P = 0.8635; Rank-Biserial correlation r = 0.04) between the two groups were not statistically significant.

**Fig. 6:**
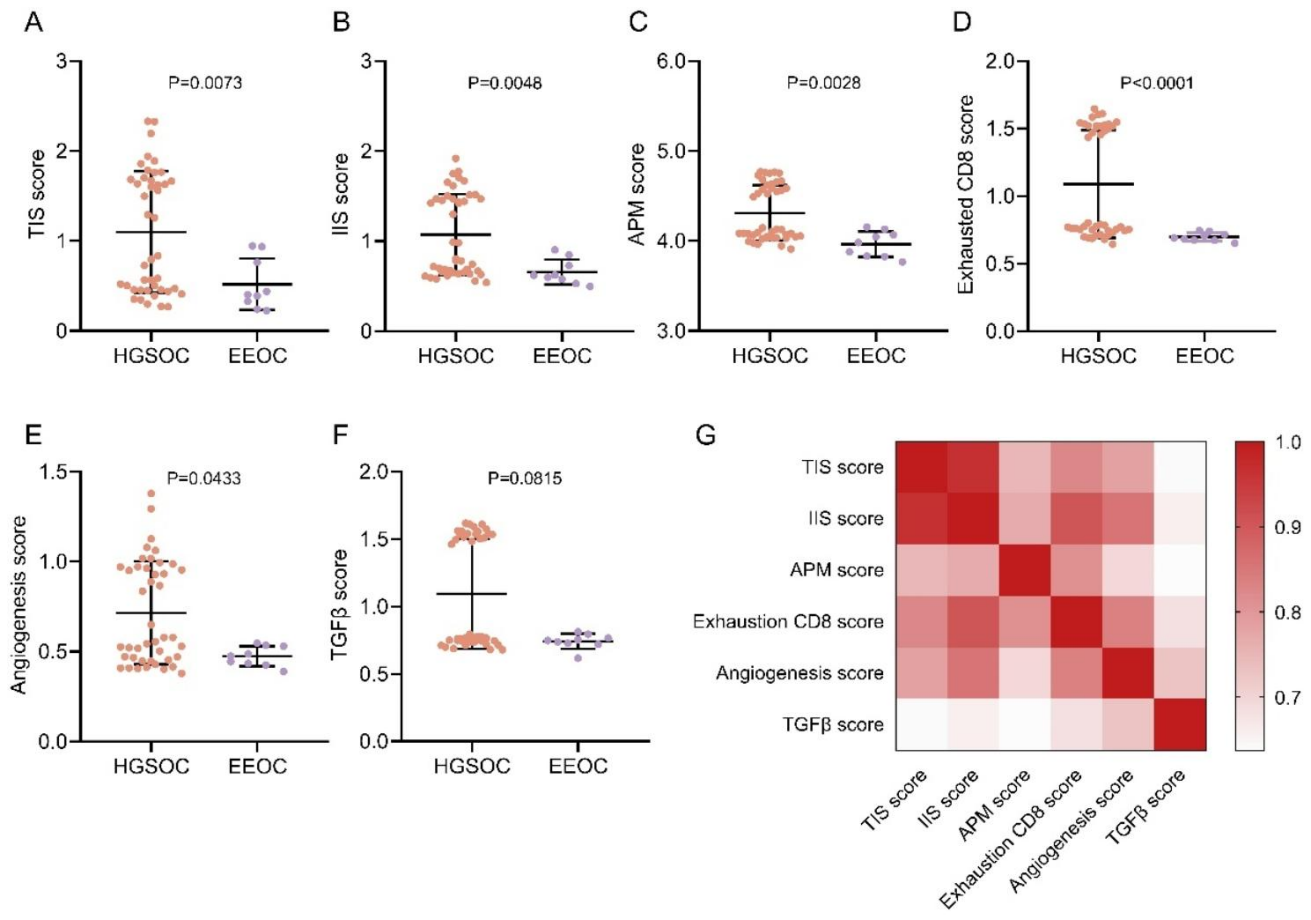
Comparison of immune-related parameters between the two subtypes. **A-F**, TIS score, IIS score, APM score, exhausted CD8 T cell score, angiogenesis score, and TGFβ score evaluated with ssGSEA methods. G, Spearman correlation coefficient matrix among six scores. HGSOC, high-grade serous ovarian cancer. EEOC, endometrioid epithelial ovarian cancer. Data was shown as ‘Mean with SD’. Statistics in A to F: Mann–Whitney U test. Correlation matrix: Spearman correlation analysis

Besides, evaluation using the ssGSEA algorithm revealed that the TIS score (range, HGSOC: 0.2687–2.3298, EEOC: 0.2254–0.9459; mean, 1.1007 vs. 0.5189; P = 0.0073; Rank-Biserial correlation r = 0.56; Fig. 6A), IIS score (range, HGSOC: 0.5408–1.9172, EEOC: 0.4979–0.9034; mean, 1.0716 vs. 0.6578; P = 0.0048; Rank-Biserial correlation r = 0.59; Fig. 6B), APM score (range, HGSOC: 3.9108–4.7688, EEOC: 3.7688–4.1541; mean, 4.3107 vs. 3.9641, P = 0.0028, Rank-Biserial correlation r = 0.62, Fig. 6C), exhausted CD8 T cell score (range, HGSOC: 0.6448–1.6448, EEOC: 0.6513–0.7446; mean, 1.0888 vs. 0.6977; P < 0.0001; Rank-Biserial correlation r = 0.78; Fig. 6D), angiogenesis score (range, HGSOC: 0.3784–1.3784, EEOC: 0.3887–0.5460; mean, 0.7148 vs. 0.4741; P = 0.0433; Rank-Biserial correlation r = 0.43; Fig. 6E) were all significantly higher in the HGSOC group compared to the EEOC group. While the mean value in TGFβ score for HGSOC was higher, no statistically significant difference was observed between these two pathological subtypes (range, HGSOC: 0.6777–1.6166, EEOC: 0.6166–0.8121; mean 1.0942 vs. 0.7416; P = 0.0815; Rank-Biserial correlation r = 0.37; Fig. 6F). The results of correlation analysis revealed that significant correlations were identified among these parameters across the cases based on the Spearman correlation analysis (all correlation coefficients r > = 0.6372, P < 0.0001, Fig. 6G).

## Discussion

Due to the overall poor prognosis of ovarian cancer, treatment for epithelial ovarian cancer often involves cytoreductive surgery combined with paclitaxel and carboplatin chemotherapy. To improve the prognosis of ovarian cancer, there is a need to explore and implement a more personalized and targeted approach. Although genetic testing is currently recommended for all ovarian cancer patients [24], the overall prognosis has not significantly improved. In this study, genetic testing was performed on two different pathological types of ovarian cancer that were often grouped together in previous clinical trials. No significant differences were observed for mutated genes between the two subtypes, which may explain why both types of pathology show sensitivity to platinum-based chemotherapy. However, the results of the mutational signature analysis revealed a significantly higher proportion of Signature 3 in HGSOC patients, suggesting a potentially higher positive rate of Homologous Recombination Deficiency (HRD) in HGSOC compared to EEOC. This suggests that patients with HGSOC may respond better to and benefit more from PARP inhibitors than patients with EEOC. Remarkably, two patients within the EEOC group were identified with pathogenic BRCA gene mutations, one exhibited a somatic BRCA2 mutation and the other one presented a germline BRCA1 mutation, suggesting a potential positive response to PARP inhibitors for these individuals. However, existing clinical trials still tend to group HGSOCs and EEOCs together [7, 25–27]. To gain a better understanding of the role of PARP inhibitors in treating ovarian cancer, future clinical trials should investigate the two histological types separately.

The understanding of the importance of immune cells within the tumor microenvironment of ovarian cancer has significantly expanded in recent years. Izar et al. utilized single cell RNA sequencing to identify immunomodulatory fibroblast sub-populations and dichotomous macrophage populations in HGSOC patients [28]. Olbrecht et al. demonstrated that TGF-β driven fibroblasts, mesothelial cells, and lymphatic endothelial cells were predictors of poor outcomes, while plasma cells were associated with favorable outcomes in HGSOC through single cell RNA sequencing [29]. Yang et al. revealed the spatial heterogeneity of infiltrating T cells in HGSOC and identified distinct immune patterns in ovarian cancer [30]. Additionally, Stur et al. uncovered substantial differences in cell cluster organization and localization within the tumour immune microenvironment of HGSOC, distinguishing poor responders from excellent responders to chemotherapy [31]. However, the majority of studies exploring the tumor immune microenvironment of OCs have predominantly focused on HGSOC due to its higher prevalence. Unfortunately, this has resulted in a limited understanding of less common histologies, such as EEOC.

Meanwhile, researchers have been exploring advanced treatment options, such as immunotherapy, to improve the prognosis of epithelial ovarian cancer. However, the response rates to immunotherapy among ovarian cancer patients remain modest, and there is currently no immunotherapy medication specifically developed for the treatment of epithelial ovarian cancer. Studies indicate that differences in the immune microenvironment contribute to varying responses to immunotherapy [32–34]. In our study, no significant differences were observed about gene mutations, TMB, and tumor heterogeneity between two histological subtypes. However, the stromal score in HGSOC was significantly higher than that of EEOC. Stromal cells play vital roles in cancer initiation and development, as well as drug resistance in various malignant tumors [35–37]. A low stromal score has been demonstrated to be a favorable factor for overall survival. No significant difference in survival was observed in our study, but it could give a hint that patients with EEOC might have a longer PFS than those with HGSOC. The higher stromal score in HGSOC compared to EEOC suggests a poorer prognosis for ovarian cancer subtypes with elevated stromal scores, which is similar with results observed in other tumors [38–40].

The response of tumors to immune checkpoint blockade (ICB) is a complex process influenced by multiple factors, including cytotoxic T cell infiltration, TMB, PD-L1 expression, and antigen presentation defects. Two mechanisms have been proposed to delineate the immunosuppressive microenvironment in tumors: one suggests that some tumors have a high infiltration of cytotoxic T cells, but these T cells tend to be exhausted and dysfunctional, while the other suggests that an overall reduction in T cells within tumors [32, 41–43].

It is well-known that T-cell exhaustion plays a major role in immune dysfunction in cancers [43]. Therefore, the local immune microenvironments of two different pathological subtypes of ovarian cancer were analyzed. We observed higher TIS score, IIS score, and CD8 T cell infiltration levels in HGSOC compared to EEOC. However, levels of exhausted CD8 T cells were significantly elevated in HGSOC. These results indicate that although HGSOC exhibits a higher level of immune cell infiltration, the proportion of exhausted immune cells is also higher. These exhausted T cells are unable to effectively eliminate tumor cells. Our results suggest that the local immune response in HGSOC may be weaker than that in EEOC. Thus, immune evasion is more likely to occur in HGSOC, and the prognosis of HGSOC is worse. Interestingly, although a higher level of CD8 T cell and exhausted CD8 T cells infiltration were observed in HGSOC than in EEOC, there was no significant difference in dysfunction score between the two subtypes. It might be caused by two main reasons for this confusing result: (a) distinct gene markers were used to evaluate dysfunction score, CD8 T cell infiltration, and exhausted CD8 T cells infiltration with different algorithms; (b) T cell dysfunction is a dynamic process, and the dysfunction score assessed by TIDE algorithm only reflects the profiles during the late stage of T cell dysfunction [21].

The second mechanism of immunosuppressive proposes a reduction of T cells in the tumor immune microenvironment, as tumor immune dysfunction and exclusion are reliable predictors of ICB response. Our results showed that TIDE scores, T cell exclusion scores, and CAF scores were significantly higher in the HGSOC group than in the EEOC group. Additionally, ssGSEA results also revealed that APM scores, exhausted CD8 T cell scores, angiogenesis scores, and TGFβ scores were also significantly higher in the HGSOC group. These findings collectively indicate that a lower presence of cytotoxic T lymphocytes within the tumor microenvironment of HGSOC, suggesting reduced tumor-killing activity in the immune environment of HGSOC. Our results provide evidence of a higher likelihood of immune evasion in patients with HGSOC, while highlighting a more favorable immune microenvironment for immunotherapy in EEOC.

Despite our interesting findings, there are several limitations in our study. Although many statistically significant different parameters were observed among two subtypes, the small number of EEOC cases remained a significant limitation. Additionally, we emphasized the transcript abundance levels as a proxy for immune cell infiltration without direct measures of immune cells. Lastly, the scores indicating potential immunotherapy outcomes have not been validated through real-world clinical trials involving EEOC. Therefore, a validation of the results in a larger cohort and biological experiments are necessary to investigate and extend the clinical relevance of our findings in the future studies.

## Conclusions

This study provides a comprehensive analysis of the immune microenvironment in HGSOC and EEOC. The high mean stromal score and more extensive infiltration and exhaustion of CD8 T cells in high-grade serous ovarian cancer indicate that high-grade serous ovarian cancer exhibits a higher level of cytotoxic T cell infiltration, yet these T cells tend to be in a dysfunctional state. Higher Tumor Immune Dysfunction and Exclusion scores, T cell exclusion scores, and CAF scores in high-grade serous ovarian cancers suggest that immune escape is more likely to occur in high-grade serous ovarian cancer, thus endometrioid ovarian cancer may be more conducive to immunotherapy. These findings contribute to a more in-depth understanding of immunotherapy application and guide future research on ovarian cancer of different pathological subtypes. Moreover, this study helps identify the ovarian cancer subtypes most suitable for immunotherapy and establishes a theoretical foundation for personalized and targeted therapy.

## Data Availability

The datasets used and/or analysed during the current study are available from the corresponding author on reasonable request.

## Abbreviations

OC: Ovarian cancer
EOC: Epithelial ovarian cancer
PFS: Progression-free survival
HGSOC: High-grade serous ovarian cancer
EEOC: Endometrioid epithelial ovarian cancer
